# Ethnobotany, chemical analysis, and antiglycation activity of *Elsholtzia* species from Northern Thailand

**DOI:** 10.3389/fphar.2025.1673250

**Published:** 2025-12-09

**Authors:** Angkhana Inta, Pitchaya Mungkornasawakul, Lalida Shank, Wittaya Pongamornkul, Yinxian Shi, Yao Fu, Ruyu Yao

**Affiliations:** 1 Yunnan International Joint Laboratory of Health Plant Resources Development, Department of Economic Plants and Biotechnology, Yunnan Key Laboratory for Wild Plant Resources, Kunming Institute of Botany, Chinese Academy of Sciences, Kunming, China; 2 Department of Biology, Faculty of Science, Chiang Mai University, Chiang Mai, Thailand; 3 Department of Chemistry and Center of Excellence for Innovation in Chemistry, Faculty of Science, Chiang Mai University, Chiang Mai, Thailand; 4 Queen Sirikit Botanic Garden, The Botanical Garden Organisation, Chiang Mai, Thailand; 5 Southeast Asia Biodiversity Research Institute, Chinese Academy of Sciences, Nay Pyi Taw, Myanmar

**Keywords:** antiglycation activity, aromatic plant, phenolic compounds, local vegetables, spices, traditional medicine

## Abstract

Seven species of *Elsholtzia* from upper northern Thailand were ethnobotanically surveyed, and the plant parts utilized by local communities were collected for chemical analysis and antiglycation activity evaluation. The 80% methanol extracts were concentrated and analyzed for total soluble phenolic content using the Folin–Ciocalteu reagent, and phytochemical profiling was performed by high-performance liquid chromatography (HPLC) against standard phenolic compounds. In addition, the extracts were investigated for antiglycation activity using the methylglyoxal (MGO)-BSA method, with aminoguanidine as a positive control. According to the ethnobotanical survey, the seven *Elsholtzia* species were traditionally used as food, medicine, and insect repellents by seven ethnic groups: Akha, Hmong, Karen, Lawa, Lisu, Tai Lue, and Tai Yai. Most species were cultivated and commonly found in agricultural areas, including upland rice fields and home gardens. HPLC analysis identified three major phenolic compounds—rosmarinic acid, ferulic acid, and luteolin—showing species- and plant part-specific variations. The leaf extract of *E. beddomei* contained the highest rosmarinic acid (667.9 mg/100 g), *E. griffithii* exhibited the highest ferulic acid (2.25 mg/100 g), and luteolin was detected exclusively in *E. stachyodes* (15.4 mg/100 g). Consistently, *E. beddomei* also had the highest total phenolic content (36.04 mg GAE/g) and the strongest antiglycation activity, with an IC_50_ value of 1.65 mg/mL. The observed trend suggests that phenolic-rich extracts, particularly those with elevated rosmarinic acid content, are strongly associated with enhanced anti-glycation potential. These findings validate local ethnobotanical practices and highlight the species-specific potential of *Elsholtzia* as sources of bioactive compounds, supporting their further development for pharmaceutical and nutraceutical applications.

## 1 Introduction

Plants are important sources for natural, nutritional and medicinal compounds (Menichetti et al., 2025; Ulian et al., 2020). Globally, the traditionally used plants are diverse, and associated ethnobotanical knowledge have inspired the discovery of useful secondary metabolites with health benefits (Leonti et al., 2017; Li and Weng, 2017). Thailand is one of the most biologically, ethnically, and culturally diverse countries, located in the Indo-Burma biodiversity hotspot (Myers et al., 2000). This diversity is particularly evident in the wealth of indigenous knowledge passed down through generations within ethnic communities. Such traditional wisdom, particularly in the field of ethnobotany, holds significant potential for scientific study and sustainable development. Previously, the rich food and medicinal plants used in Thailand have been reported (Inta et al., 2025). However, much of this knowledge is at risk of being lost due to socio-economic changes and the lack of systematic documentation (Yao et al., 2024). Preserving and exploring this knowledge are expected to lead to valuable contributions in fields such as medicine, nutrition, and economic development (Gao, 2024; Yao et al., 2021).

The genus *Elsholtzia* belongs to the tribe Elsholtzieae, subfamily Nepetoideae, and family Lamiaceae. It comprises approximately 40 species worldwide, most of which are naturally distributed in the temperate and tropical regions of Asia. Some species are cultivated in Europe and North America. In Southeast Asia, seven species have been reported in Vietnam, five in the Indochina Peninsula, eleven in Myanmar and three in Malaysia. In Thailand, eight species of *Elsholtzia* have been recorded, with most distributed in the northern region of the country, particularly in the open forests or forest edges at elevations starting from 600 m above sea level. Most of these species have also been cultivated by local people (Bongcheewin et al., 2015).

This notable group of plants have remarkable chemical properties and diverse applications in food and traditional medicine in different countries. For instance, in China, 14 species of *Elsholtzia* are used as herbal medicine, among which at least four species are also used for culinary and tea: *E. kachinensis*, *E. communis*, *E. bodinieri* and *E. penduliflora*, and eight species are used as aromatic plants (according to the Scientific Database of China Plant Species https://db.kib.ac.cn/). In India, *Elsholtzia densa* and *E. eriostachya* are used as culinary herbs (Chen et al., 2022), while in Vietnam, the Hmong and Dao ethnic groups utilize essential oils from *E. penduliflora* and *E. blanda* in traditional medicine for treating fever, pain, and insect bites (Dung et al., 2023). These essential oils are also incorporated into soap and spa products, demonstrating their commercial potential. *Elsholtzia* is also important sources in traditional Chinese medicine to treat cold, fever, pneumonia, etc. (Chen et al., 2022). *Elsholtzia* species are well known for their essential oil composition, which includes bioactive compounds with pharmacological properties. Studies have identified key components such as perillene, elsholtzia ketone, α-dehydro-elsholtzione, dehydro-elsholtzia ketone, and humulene (Korolyuk et al., 2002).

Beyond these applications, *Elsholtzia* species may have significant health benefits, particularly in the prevention of diabetes-related complications. Diabetes is a metabolic disorder characterized by impaired glucose regulation and insulin dysfunction, leading to elevated blood sugar levels. High blood sugar is a condition in diabetes which causes free radicals from oxygen leading to various side effects. Glycation is a non-enzymatic reaction between proteins in the body with excess sugar producing advanced glycation end-products (AGEs) which can deposit slowly in the tissues causing structural changes and disfunction. Prolonged hyperglycemia contributes to the formation of AGEs, which play a crucial role in the development of complications such as cardiovascular disease, kidney failure, and retinal damage. AGEs also trigger inflammatory responses through their interaction with receptors (RAGEs), further exacerbating disease progression. Recent studies have indicated that plant-derived phytochemicals, particularly phenolic compounds and flavonoids, exhibit antiglycation properties (Kaewnarin et al., 2013). Bioactive compounds such as procyanidins from cinnamon, caffeic acid and chlorogenic acid from tea, and luteolin, apigenin, and rosmarinic acid from Lamiaceae species have demonstrated potential in mitigating glycation-related damage (Kaewnarin et al., 2013). Given the rich phytochemical diversity of *Elsholtzia*, it is essential to explore their potential antiglycation activity and medicinal applications.

Despite the recognized potential, comprehensive studies on *Elsholtzia* species in Thailand remain limited; many plants in this genus are used by various ethnic groups for their aromatic and medicinal properties, yet their utilization is not widespread. Understanding the distribution and traditional uses of these plants in Thailand can provide insights into their potential development as functional foods and medicinal crops, particularly for health-conscious consumers. Furthermore, these plants may serve as an economic resource for local communities, promoting biodiversity conservation and sustainable agricultural practices.

This study combined an ethnobotanical survey of seven *Elsholtzia* species used by local ethnic communities in upper northern Thailand with chemical analysis of the collected plant parts, including total phenolic content and HPLC profiling, and evaluated their antiglycation activity using the methylglyoxal (MGO)-BSA assay. To the best of our knowledge, this study presents the first evidence of antiglycation activity and quantification of key bioactive compounds (rosmarinic acid, ferulic acid, and luteolin) in *Elsholtzia beddomei*, *E. stachyodes*, *E. communis*, *E. blanda*, and *E. griffithii*, and further records the ethnobotanical uses of *Elsholtzia* species in Northern Thailand. By bridging traditional knowledge with scientific validation, this research contributes to the conservation and sustainable utilization of *Elsholtzia* species, while also supporting their potential development as economic crops that promote community wellbeing and health-conscious consumption.

## 2 Materials and methods

### 2.1 Study sites

**FIGURE 1:**
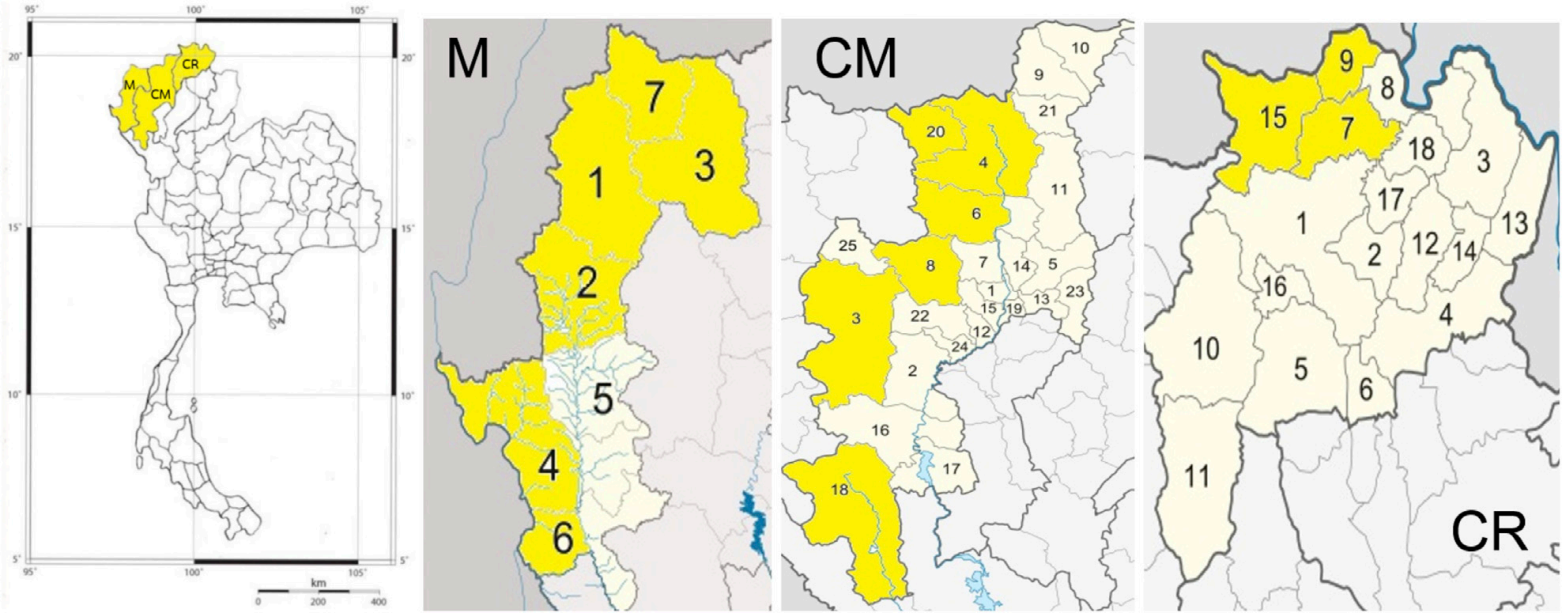
The 15 districts across three provinces selected as study sites in upper northern Thailand. [Including Mueng Mae Hong Son (1), Mae Sariang (2), Mae Lanoi (3), Sob Mei (4), Khun Yuam (6) and Pang Ma Pha (7) districts in Mae Hong Son Province (M), Mae Chaem (3), Chiang Dao (4), Mae Taeng (6), Samoeng (8), Omkoi (18) and Wiang Haeng (20) districts in Chiang Mai Province (CM), Mae Chan (7), Mae Sai (9) and Mae Fa Luang (15) districts in Chiang Rai Province (CR)].

The study was conducted across 40 villages representing seven ethnic groups: Akha, Hmong, Karen, Lua, Lisu, Tai Lue, and Tai Yai. These villages are located in 15 districts across three provinces in upper northern Thailand—six districts in Chiang Mai, three in Chiang Rai, and six in Mae Hong Son (Figure 1). The study sites featured predominantly mixed deciduous forest, dipterocarp forest, and dry evergreen forest. Elevation ranged from 345 to 1,800 m above sea level. Despite relocating to new habitats, the communities have preserved their traditional knowledge and way of life.

### 2.2 Ethnobotanical investigation

The ethnobotanical investigation in this study was carried out during 2020–2022 with a focus on an understanding of the local knowledge and practices related to *Elsholtzia* species. The process began by explaining the objectives of the study to the village head, who then advised the key informants on how to collect information from other experienced villagers using a snowball sampling approach. The information gathering process was conducted using a combination of free-listing, semi-structured interviews, group discussion and the walk-to-the-wood method. The questions were designed to collect data on the (i) vernacular name of the plants, (ii) parts used, (iii) methods for preparation, and (iv) characteristics of the plant material.

A total of 80 key informants, aged between 25 and 75 years, participated in the study. They included farmers, local healers, and merchants. The free-listing method was used to gather an initial list of plants used by the informants during group discussion. This was followed by semi-structured interviews, which allowed for a more in-depth exploration of the plants, including the parts used, preparation methods, and purposes of use. The walk-to-the-wood method involved accompanying the informants as they visited their home gardens, nearby forests, and other areas to collect specimens of the plants discussed. This hands-on approach provided an opportunity to observe the plants in their natural habitats and gather additional information from the informants. All participants provided informed consent prior to their participation in the study. The participants were fully informed about the purposes and procedures of the study and were free to withdraw at any time. Those who were literate provided written consent, while those who were illiterate provided verbal consent with a witness present.

### 2.3 Plant identification

The *Elsholtzia* specimens collected during the field survey were deposited as voucher specimens in the Herbarium of Queen Sirikit Botanic Garden (QBG) and were accompanied by photographs taken during the survey. The accepted scientific names and families of the identified plant species were determined based on the Plants of the World Online Database (powo.science.kew.org), which is a comprehensive database that provides information on the current accepted name and family for plant species. In some cases, living specimens were collected to ensure future accurate identification and facilitate propagation efforts.

### 2.4 Chemical analysis

Although seven *Elsholtzia* species were documented during the ethnobotanical, with some samples collected in 2023, chemical analyses were conducted only on species for which sufficient and high-quality plant material was available. Limited specimen availability due to seasonal constraints, small or fragmented populations, and the necessity of preserving voucher specimens precluded the inclusion of some species. Additionally, time and budgetary constraints restricted the number of samples processed, resulting in five species being selected for chemical analysis. The collected plant materials — *Elsholtzia beddomei* (flowers and leaves), *E. stachyodes* (flowers), *E. communis* (flowers), *E. blanda* (leaves), *E. griffithii* (flowers) — were air dried at room temperature (25 °C ± 2 °C). Each dried plant samples (2.00 g) was accurately weighed and transferred into a pear-shaped flask. The sample was extracted with 100 mL of 80% methanol, sealed, and left to stand overnight at room temperature (25 °C ± 2 °C). The mixture was subsequently subjected to ultrasonication for 60 min and filtered through Whatman No. 1 filter paper. The filtrate was concentrated under reduced pressure using a rotary evaporator and adjusted to a final volume of 5 mL with 80% methanol. The extract was stored at 4 °C until further analysis. The total soluble phenolic content was determined using the Folin–Ciocalteu reagent following a modified method of Thitilertdecha et al. (2008).

Approximately 0.100 g of the extract was accurately weighed and adjusted to a final volume of 1 mL with 80% methanol. The solution was filtered through a 0.45 μm nylon syringe filter prior to analysis. HPLC analysis was performed using a system equipped with a quaternary pump and a UV detector set at 280 nm. Separation was achieved on an Eclipse C18 column (4.6 × 150 mm, 5 μm) with a guard column, at a flow rate of 0.8 mL/min and an injection volume of 20 μL. The mobile phase consisted of solvent A (0.1% formic acid in water) and solvent B (100% acetonitrile) using the following gradient program: 0–10 min, 94% A and 6% B; 10–50 min, 70% A and 30% B; 50–60 min, 50% A and 50% B. Standard compounds—rosmarinic acid, luteolin, apigenin (Sigma-Aldrich), and ferulic acid (Fluka)—were used for the identification and quantification of phenolic compounds in the extracts. Data were expressed as mean ± standard deviation (n = 3) and subjected to statistical analysis.

### 2.5 Antiglycation activity testing

The inhibition of glycation reactions by *Elsholtzia* plant extracts was evaluated using the MGO-BSA method, which was chosen for its established reliability, reproducibility, and physiological relevance in modeling glycation processes. Although alternative techniques, such as fluorometric assays and HPLC–MS/MS, offer higher analytical efficiency, their use was limited by instrument accessibility during the study period. Future investigations will incorporate these advanced methodologies to further substantiate the MGO-BSA–based results (Kaewnarin et al., 2014; Peng et al., 2007). A volume of 300 µL of a mixed solution of BSA (Bovine Serum Albumin) at a concentration of 10 mg/mL was pipetted into a screw tube. Subsequently, 30 µL of MGO (methylglyoxal) at a concentration of 0.5 M was added. Sample extracts at various concentrations were then added, and the final volume was adjusted to 3 mL. The mixture was incubated at 37 °C for 7 days. After incubation, the fluorescence intensity was measured using a luminescence microplate reader (excitation at 370 nm; emission at 440 nm), and the percentage inhibition of the glycation reaction was calculated. Aminoguanidine (AG) was used as the positive control.

$$\%\;inhibition=\frac{\left(\text{intensity}\;\text{of}\;\text{control}-\text{intensity}\;\text{of}\;\text{sample}\right)}{\text{intensity}\;\text{of}\;\text{control}}\;x\;100$$

The percentage inhibition values at different concentrations were plotted to generate a dose–response curve, from which the 50% inhibitory concentration (IC_50_) was calculated.

## 3 Results

### 3.1 Surveyed *Elsholtzia* species and associated traditional knowledge

**FIGURE 2:**
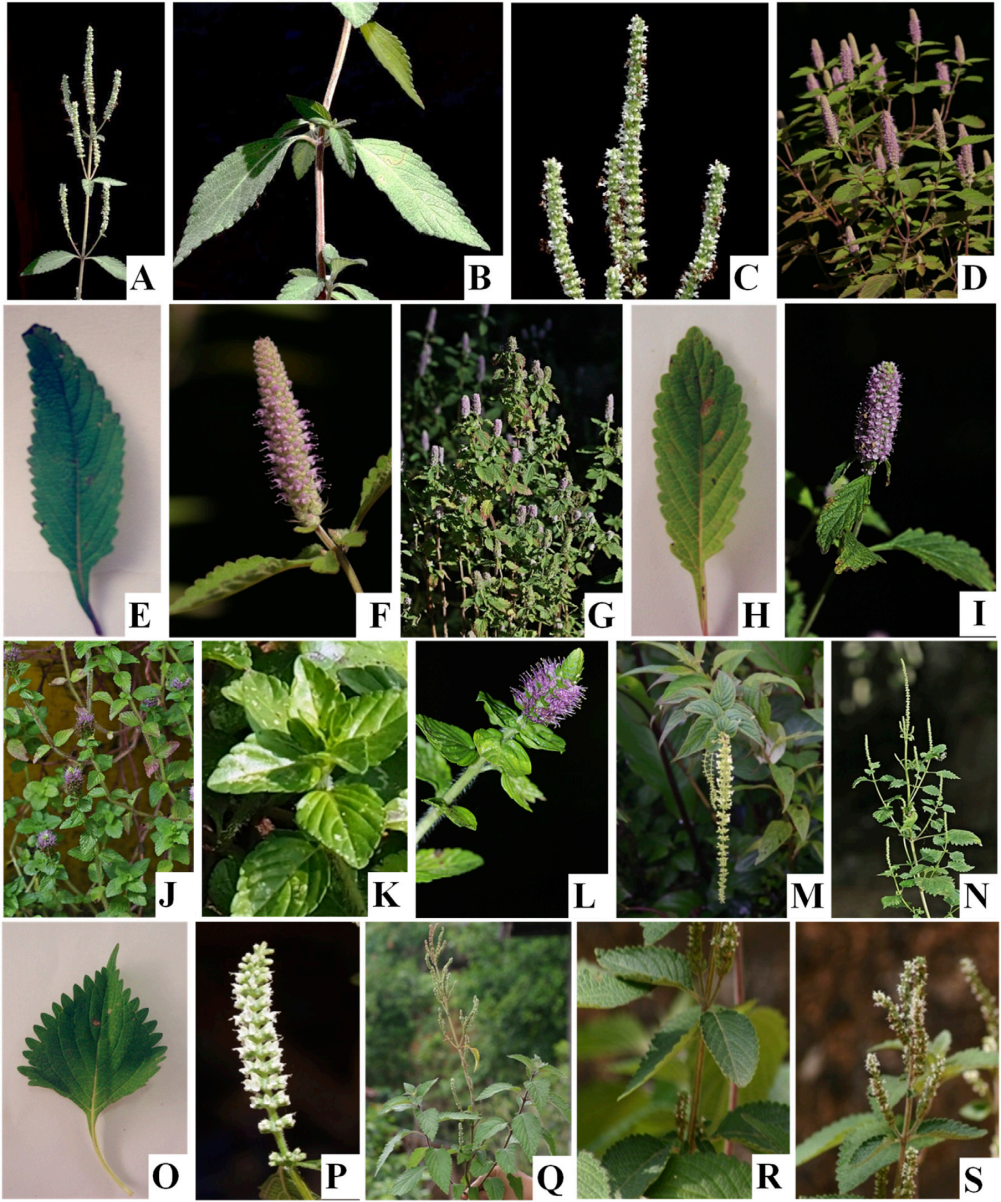
Morphological characters of *Elsholtzia* species used by ethnic groups in upper northernThailand. **(A–C)** *E. beddomei* C.B.Clarke ex Hook.f.; **(D–F)** *E. communis* (Collectt & Hemsl.) Diels; **(G–I)** *E. griffithii* Hook.f.; **(J–L)** *E. kachinensis* Prain; **(M)** *E. penduliflora* W.W.Sm.; **(N–P)** *E. stachyodes* (Link) Raizada & Saxena; **(Q–S)** *E. winitiana* Craib.

Seven *Elsholtzia* species were used by ethnic groups in upper northern Thailand (Table 1; Figure 2). Most of *Elsholtzia* species was used for food as spice and vegetable. *Elsholtzia penduliflora* and *E. winitiana* was used as medicine for Hmong and Akha ethnic groups, respectively. Both *E. beddomei* and *E. stachyodes* were used as an insect repellent in barns. *Elsholtzia kachinensis* was widely used by most of the ethnic groups including Tai Yai, Tai Lue, Akha, and Lisu. Most *Elsholtzia* species were cultivated plants commonly found in agricultural areas, including upland rice fields and home gardens. Only *E. beddomei* was found in natural habitats (Table 1).

**TABLE 1:** Ethnobotanical use of seven *Elsholtzia* species by ethnic groups in upper northern Thailand.

Species [voucher specimen no.]	Ethnic group	Vernacular name	Source			Use category	Part used: How to use
			Rice field	Home garden	Forest		
*Elsholtzia beddomei* C.B.Clarke ex Hook.f. [WP5703]	Karen	Hor Prei Pa			✓	insect repellent	Whole plant: used fresh or dried as an insect repellent in barns
^a^ *Elsholtzia communis* (Collectt & Hemsl.) Diels [WP7634]	Karen	Hor Wor	✓			food	Shoot and inflorescences: used fresh or dried as a spice to create the signature taste and aroma in local large cucumber soup or chili paste; fresh parts are sold in local or roadside markets
	Lua	Hlan	✓			food	Shoot and inflorescences: used fresh or dried as a spice to create the signature taste and aroma in local large cucumber soup or chili paste; consumed fresh as a side-dish vegetable
	Akha	Yia Pi La Chong	✓			food	Shoot and inflorescences: used fresh as a spice to create the signature taste and aroma in fermented bamboo soup or chili paste
^a^ *Elsholtzia griffithii* Hook.f. [WP6522]	Tai Yai	Lum Pum	✓	✓		food	Shoot: used fresh as a vegetable in local bean soup; consumed fresh as a side-dish vegetable
	Lisu	Lum Pum	✓			food	Shoot: used fresh as a vegetable in local pumpkin soup; consumed fresh as a side-dish vegetable
^a^ *Elsholtzia kachinensis* Prain [WP6527]	Tai Yai	Hern	✓	✓		food	Shoot: used fresh as a vegetable in bone soup; consumed fresh as a side-dish vegetable
	Tai Lue	Lern	✓	✓		food	Shoot: used fresh as a vegetable in bone soup; consumed fresh as a side-dish vegetable; fresh parts are sold in local or roadside markets
	Akha	Lor Chor	✓			food	Shoot: used fresh as a vegetable in bone soup; consumed fresh as a side-dish vegetable
	Lisu	Xiao Chai	✓			food	Shoot: used fresh as a vegetable in bone soup; consumed fresh as a side-dish vegetable
*Elsholtzia penduliflora* W.W.Sm. [WP2256, WP2209]	Hmong	Yan Chua Tou		✓		medicine	Leaves: used fresh as a spice in chicken soup consumed for body nourishment; decoction used for bathing to promote good health
^a^ *Elsholtzia stachyodes* (Link) Raizada & Saxena [WP8235]	Tai Yai	Han	✓	✓		food	Shoot: used fresh as a vegetable
	Karen	Hor Prei		✓		food insect repellent	Shoot: used fresh as a spice for eliminating fishy smell from offal soup; consumed fresh as a side-dish vegetable Whole plant: used fresh or dried as an insect repellent in barns
*Elsholtzia winitiana* Craib [WP6476]	Akha	Ngu Shee		✓		medicine	Leaves: decoction used for treating cancer, food poisoning, and dog bite (Inta et al., 2008)

^a^ The *Elsholtzia* species was sold in local and roadside markets.

### 3.2 Chemical composition of surveyed *Elsholtzia* species

**FIGURE 3:**
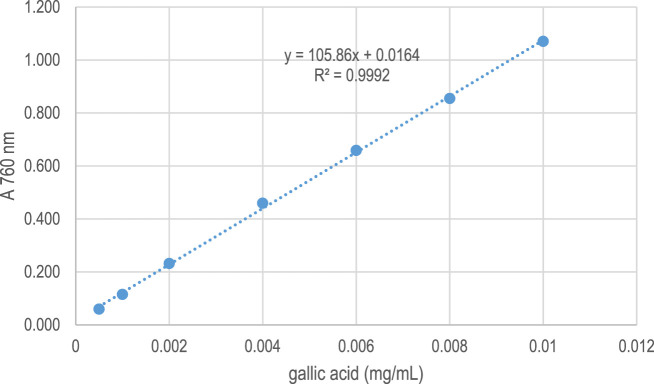
Standard curve of gallic acid at various concentrations for the determination of total soluble phenolic compounds.

The total soluble phenolic content of extracts from the five *Elsholtzia* species collected from upper northern Thailand was analyzed. The extracts were reacted with Folin-Ciocalteu reagent, and the absorbance was measured at 760 nm. The values were compared against a standard curve of gallic acid (R^2^ = 0.9992) (Figure 3), and the results are presented in Table 2. It was found that the total soluble phenolic content ranged from 6.50 to 36.04 mg of gallic acid equivalents per gram of sample (mg GAE/g sample). The highest phenolic content, 36.04 mg GAE/g sample, was observed in the extract from *E. beddomei*, which was obtained from the leaves. The lowest content, 6.50 mg GAE/g sample, was detected in the extract from the *E. communis*.

**TABLE 2:** The phenolic compounds in five *Elsholtzia* species from upper northern Thailand.

Species	Sample parts	Content of the phenolic compounds^a^			
		Total phenolics mg GAE/g sample	Rosmarinic acid (RA) mg/100 g sample	Ferulic acid (FA) mg/100 g sample	Luteolin (LU) mg/100 g sample
*Elsholtzia beddomei*	flowers	15.08 ± 1.13^b^	-	0.652 ± 0.1	-
	leaves	36.04 ± 1.06^a^	667.9 ± 8.7	-	-
*Elsholtzia stachyodes*	flowers	13.65 ± 0.11^c^	-	-	15.4 ± 0.8
*Elsholtzia communis*	flowers	6.50 ± 057^e^	23.5 ± 0.6	-	-
*Elsholtzia blanda*	leaves	8.44 ± 0.02^d^	-	-	-
*Elsholtzia griffithii*	flowers	14.51 ± 0.07^c^	36.7 ± 0.1	2.25 ± 0.1	-

^a^ The mg GAE/g sample refers to milligrams of gallic acid equivalents per gram of crude extract. Experimental results are reported as mean ± standard deviation (n = 3). The ^a–e^ indicated statistically significant differences at P < 0.05.

In *E. beddomei*, which had the highest total phenolic content, a comparison between the flower and leaf parts revealed that the leaves contained more than twice the amount of soluble phenolic compounds compared to that from the flowers of the same species.

**FIGURE 4:**
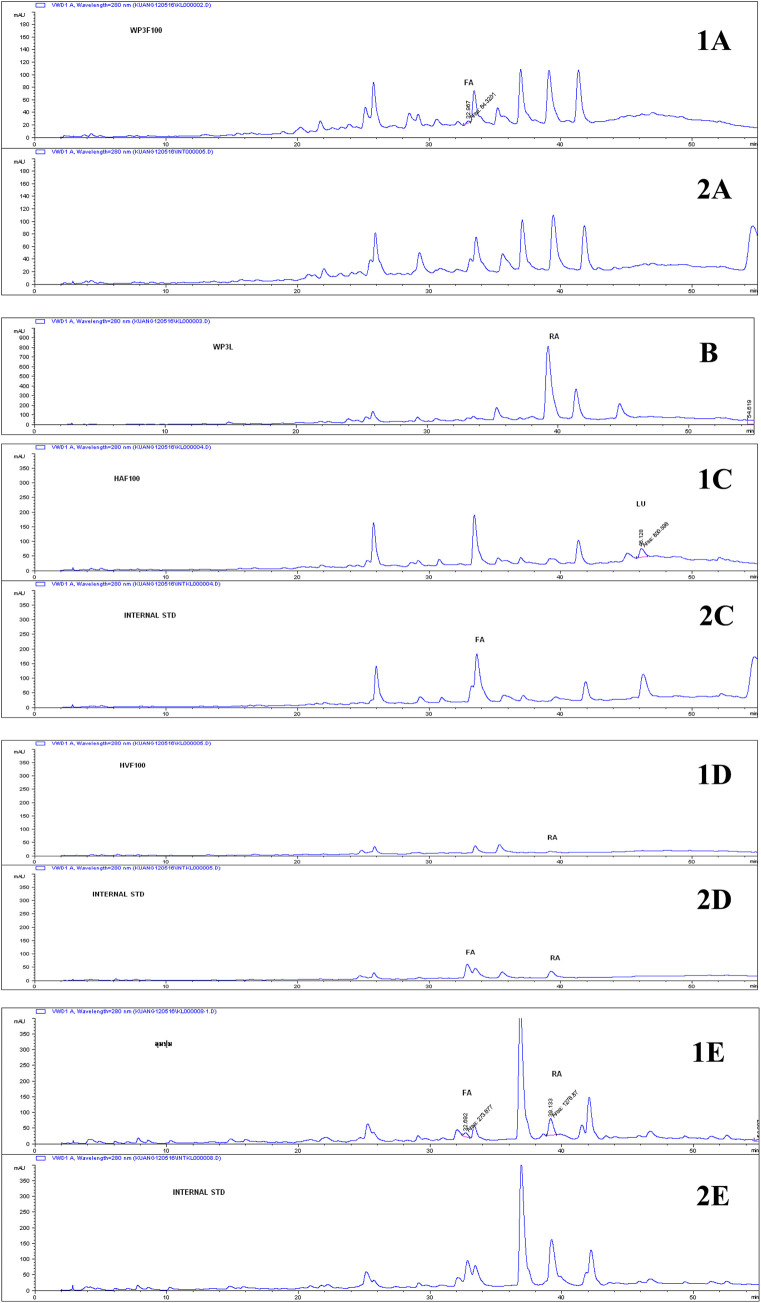
Chromatograms of *Elsholtzia* species extracts (1) and chromatograms with internal standards (2) including *Elsholtzia beddomei* (A, flowers; B, leaves), *Elsholtzia stachyodes* (C), *Elsholtzia communis* (D), *Elsholtzia griffithii* (E)

The phytochemical content from the five *Elsholtzia* species was analyzed using high-performance liquid chromatography (HPLC) and compared with the standard phenolic compounds (Table 2; Figure 4). Four phenolic compounds were identified: rosmarinic acid (RA), ferulic acid (FA), and luteolin (LU). The highest amount of rosmarinic acid, 667.9 mg per 100 g of sample, was detected in the leaf extract of *E. beddomei*. The highest ferulic acid content, 2.25 mg per 100 g of sample, was observed in the *E. griffithii*. Luteolin was detected only in the *E. stachyodes* (15.4 mg per 100 g of sample).

### 3.3 Antiglycation activity of surveyed *Elsholtzia* species

According to the experimental results in Table 3, the leaf extract of *Elsholtzia beddomei* showed the highest antiglycation activity in the MGO-BSA system, with an IC_50_ value of 1.65 mg/mL. In contrast, the leaf extract of *E. blanda* exhibited the lowest antiglycation activity, with an IC_50_ value of 8.10 mg/mL. However, the effectiveness of the extracts in this group was still lower when compared to the standard inhibitor, aminoguanidine, with the IC_50_ value of 0.29 mg/mL.

**TABLE 3:** *In vitro* antiglycation activity of the 80% methanol extracts of *Elsholtzia* species in the MGO-BSA system.

Species	Sample part	Antiglycation activity IC_50_ (mg/mL)^a^
*Elsholtzia beddomei*	flowers	3.46 ± 0.15^c^
	leaves	1.65 ± 0.10^a^
*Elsholtzia stachyodes*	flowers	2.97 ± 0.17^b^
*Elsholtzia communis*	flowers	5.21 ± 0.05^d^
*Elsholtzia blanda*	leaves	8.10 ± 0.31^e^
*Elsholtzia griffithii*	flowers	3.72 ± 0.17^c^
Standard inhibitor		
Aminoguanidine		0.29 ± 0.02

^a^ The experimental results are reported as mean ± standard deviation.

The ^a–e^ indicates statistically significant differences at P < 0.05.

## 4 Discussion

### 4.1 Ethnobotanical uses of *Elsholtzia* species in upper northern Thailand and other Asian countries

Seven species of *Elsholtzia* were found to be used by ethnic groups in upper northern Thailand, namely, *E. griffithii*, *E. kachinensis*, *E. winitiana*, *E. penduliflora*, *E. communis*, *E. beddomei*, and *E. stachyodes*. Among these, *E. communis* was not previously recorded as part of the *Elsholtzia* (Lamiaceae) flora in Thailand. *E. blanda* was encountered during our field surveys in upper northern Thailand; however, from our ethnobotanical interview it has not been reported as being used by local ethnic groups. *Elsholtzia pilosa* was not found in this study. It has only been collected once, in 1922, and appeared to have disappeared from natural habitats in Thailand (Bongcheewin et al., 2015). Most *Elsholtzia* species documented in this study were found in cultivated areas, such as rice fields and homegardens. Only *E. beddomei* was observed along forest margins.

*Elsholtzia communis* was identified as the most culturally significant seasonal spice among the Karen and Lua peoples (Brikut and Inta, 2019; Inta et al., 2025; Moonjai and Inta, 2016; Panyadee et al., 2021). This species was traditionally planted by nearly every household alongside their rotational upland rice fields each year. It was harvested throughout its growth cycle, from the vegetative to reproductive stages, directly from rice fields for culinary use. Fresh or dried leaves and inflorescences were commonly used as a spice to impart a distinctive taste and aroma to local dishes, such as large cucumber soup and chili paste, or consumed fresh as a side-dish vegetable. Mature dried inflorescences, containing tiny seeds, were typically harvested around November and stored above the cooking stove for use as planting material in the following season. The *E. communis* has been traditionally used for both medicinal and culinary purposes in Myanmar, Vietnam, and China. Medicinally, it is particularly employed in the treatment of respiratory ailments such as tonsillitis, fever, and cough, as well as for conditions including nosebleeds and menstrual disorders (Chen et al., 2022).

*Elsholtzia kachinensis* was the most popular among the ethnic groups in Northern Thailand including Tai Yai, Tai Lue, Akha, and Lisu. Fresh shoot was used as a vegetable in bone soup or consumed fresh as a side-dish vegetable. The *E. kachinensis* was planted a small plot in home gardens. It was planted in a big plot in the agricultural area with other commercial vegetables by some household for harvesting and selling in local or roadside markets. It can be propagated by root cutting. The *E. kachinensis* has traditionally been used for culinary purposes in Myanmar and Southern China (Bongcheewin et al., 2015).

*Elsholtzia penduliflora* was specifically used for traditional medicine only in the Hmong communities in upper northern Thailand (Nguanchoo et al., 2022). Fresh leaves were used as a spice in chicken soup consumed for body nourishment or preparing decoction used for bathing to promote good health. The *E. penduliflora* was also used as traditional medicine by Hmong in Southern China (Chen et al., 2022). It seems that when Hmong people migrated to upper northern Thailand, they carried the *E. pendulifkora* with them and planted in their home gardens. The Hmong in Honghe Prefecture in Yunnan Province, southern China used the *E. penduliflora* for bronchitis, influenza, cancer, and human infertility (Chen et al., 2022).

*Elsholtzia winitiana* was found in open places in hill evergreen, deciduous, pine or dipterocarp forests of upper northern Thailand whereas in our study it was found in Akha home garden (Bongcheewin et al., 2015; Inta et al., 2008). The *E. winitiana* was used as traditional medicine by local Akha healers for treating cancer, food poisoning, and dog bite (Inta et al., 2008). The Akha local healers collected this plant and planting it in their home gardens for immediate access. The *E. winitiana* was used as traditional medicine for treating measles, headache, and cold by Akha or Hani people around the Naban River Watershed National Nature Reserve, China (Chen et al., 2022). The *E. winitiana* seems to be a cultural medicinal plant of Akha group.

Among different biocultural backgrounds, ethnobotanical knowledge varies, although using plants as food and medicine is a general tradition (Yao et al., 2023). For example, even in the same ecosystem, people of two cultural backgrounds have different ethnobotanical knowledge (Quave and Pieroni, 2015), while the cross-border circulation of local knowledge facilitates the conservation and utilization of such plants and related traditional knowledge (Prakofjewa et al., 2023). The present work reported the traditional uses of *Elsholtzia* species in upper northern Thailand, showing high diversity in plant species and usages. While this genus is widely distributed in its neighbor regions such as the Great Mekong Subregion (Chen et al., 2022), where the cultural similarity also exists, it is worthy of exploring the traditional knowledge of *Elsholtzia* species in a perspective of comparative ethnobotany.

### 4.2 Chemical composition of some *Elsholtzia* species used by ethnic groups in upper northern Thailand

The phenolic content observed in *Elsholtzia* species studied—particularly in *E. beddomei*—is notably high when compared with several commonly consumed vegetables and medicinal plants. For instance, the total phenolic content in spinach (*Spinacia oleracea*) and broccoli (*Brassica oleracea* var. *italica*), which are known for their antioxidant properties, typically ranges from 5 to 15 mg GAE/g in dried samples (Chu et al., 2002; Zhou and Yu, 2006). In this context, the 36.04 mg GAE/g sample found in *E. beddomei* leaf extract is more than double the phenolic levels in these vegetables, highlighting its potential as a rich source of natural antioxidants. Furthermore, the phenolic content of *Camellia sinensis* (green tea) leaves, one of the most studied medicinal plants, often falls within the range of 20–30 mg GAE/g, depending on processing and cultivar (Balentine et al., 1997; Yen and Chen, 1995). This comparison underscores the significant phenolic richness of *E. beddomei*, making it comparable to or even surpassing established sources of dietary antioxidants. These findings support the ethnobotanical knowledge of local communities in Northern Thailand and point toward the potential for developing *Elsholtzia* species, especially *E. beddomei*, as functional food ingredients or phytopharmaceutical candidates (Balasundram et al., 2006). However, it should be noted that the chemical composition and yield of phenolic compounds can be affected by multiple factors, including the choice of extraction solvent, the extraction method, and variability in plant material (Pudziuvelyte et al., 2018).

The variation in total phenolic content between different plant parts, such as leaves and flowers, can be attributed to their distinct physiological roles and exposure to environmental stressors (Lattanzio et al., 2006). In the *E. beddomei*, which had the highest total phenolic content, a comparison between the flower and leaf parts revealed that the leaves contained more than double the amount of soluble phenolic compounds compared to that from the flowers of the same species (Balasundram et al., 2006; Sriraksa and Seekhaw, 2024). This difference is likely due to the fact that leaves are the primary site of photosynthesis and are more frequently exposed to ultraviolet (UV) radiation, herbivores, and pathogens. As a result, they tend to accumulate higher levels of phenolic compounds, which function as protective agents against oxidative stress and biotic threats. In contrast, flowers, which have more specialized roles in reproduction, often contain different classes of secondary metabolites such as pigments (e.g., anthocyanins) and volatile compounds that attract pollinators rather than serve as strong antioxidants (Harborne and Williams, 2000). Additionally, biosynthetic pathways for phenolics may be more active in leaf tissues due to greater metabolic activity (Treutter, 2005). These findings highlight the importance of selecting specific plant parts when evaluating phytochemical content and bioactivity, particularly in medicinal and functional plant research.

Three phenolic compounds were identified in the studied *Elsholtzia* species: rosmarinic acid, ferulic acid, and luteolin. Among them, the highest concentration of rosmarinic acid was detected in the leaf extract of *E. beddomei*, supporting its strong antioxidant potential (Alagawany et al., 2021; Petersen and Simmonds, 2003). Ferulic acid was found in the highest amount in *E. griffithii* (Ou and Kwok, 2004; Zhao et al., 2021), while luteolin was uniquely detected only in *E. stachyodes* (Lin et al., 2008; Seelinger et al., 2008). These compounds are well-known secondary metabolites in the Lamiaceae family and are associated with various pharmacological activities, including antioxidant, anti-inflammatory, antimicrobial, and anticancer properties. Their presence reinforces the therapeutic potential of these *Elsholtzia* species and aligns with findings from other members of the Lamiaceae family (Kaewnarin et al., 2013). For example, rosmarinic acid is commonly found in high levels in *Rosmarinus officinalis* and *Ocimum basilicum*, and has been widely studied for its protective effects against oxidative stress and chronic diseases. Ferulic acid, typically present in plant cell walls, contributes to UV protection and pathogen resistance, while luteolin is a flavonoid known for its strong anti-inflammatory and neuroprotective effects.

The selective distribution of these compounds among species suggests that phytochemical composition may vary not only between species but also according to environmental, genetic, and developmental factors. The application of advanced chromatographic techniques, particularly high-performance liquid chromatography (HPLC), enables accurate quantification and profiling of such bioactive compounds. Future studies could employ HPLC in combination with mass spectrometry (LC-MS/MS) (Cuyckens and Claeys, 2004) or metabolomics approaches to monitor chemical variation over time, across habitats, and during different plant growth stages. Such analyses are essential for quality control, pharmacological standardization, and valorization of *Elsholtzia* species in ethnomedicine and potential functional food development.

### 4.3 Exploring the antiglycation potential of *Elsholtzia* species traditionally used by ethnic communities in upper northern Thailand

Local *Elsholtzia* species are rich in phytochemicals with demonstrable biological activities, including the inhibition of protein glycation—a key mechanism underlying many diabetes-related complications. In our study, the crude leaf extract of *E. beddomei* exhibited the strongest antiglycation activity among the species analyzed, though it was less potent than the benchmark inhibitor aminoguanidine. Specifically, the IC_50_ value of aminoguanidine is approximately 0.29 mg/mL (Cushnie et al., 2024; Lee et al., 2017) whereas our crude extract displayed higher (i.e., weaker) IC_50_ values. This is likely due to the complex matrix of inactive or less active compounds present in crude extracts. Our observation aligns with numerous studies demonstrating that crude plant extracts often show lower antiglycation efficacy compared to standard inhibitors, but significantly improve upon purification, which concentrates active phenolic constituents.

Furthermore, there is a strong and consistent correlation between total phenolic content and antiglycation activity in plant extracts. Research on various edible and medicinal plants—including *Punica granatum*, *Mangifera indica*, and *Dimocarpus longan*—has shown that extracts with higher phenolic levels demonstrate stronger inhibitory activity in methylglyoxal-BSA assays, often outperforming aminoguanidine under similar testing conditions (Kaewnarin et al., 2013; Tsai and Lin, 2019). Likewise, investigations into spices and herbs have confirmed that total phenolic content is highly correlated (r = 0.84 in BSA-MGO assays) with antiglycation activity (Xie and Chen, 2019).

Given that *E. beddomei* showed both the highest phenolic content (36.04 mg GAE/g sample) and the strongest antiglycation activity among the tested *Elsholtzia* species, our data reinforce the hypothesis that glycation inhibition efficacy is tied to phenolic compound abundance. Future steps such as bioassay-guided fractionation and purification could isolate the specific phenolics driving this activity, potentially yielding compounds with IC_50_ values comparable to or better than aminoguanidine.

If *Elsholtzia* extracts can eventually be used as substitutes for the standard synthetic inhibitor aminoguanidine (commercially known as Pimagedine®), which is currently used to treat complications of diabetes, it could help reduce the side effects associated with synthetic drugs. Aminoguanidine has been reported to cause adverse effects such as fever, chills, muscle weakness, gastrointestinal issues, anemia and in some cases, vitamin B6 deficiency (Reddy and Beyaz, 2006).

In this study, we focused specifically on the antiglycation activity of *Elsholtzia* species to provide a targeted investigation relevant to potential functional food and phytopharmaceutical applications. Owing to time and budget constraints, other biological activities, including antioxidant, anticancer (Hoang et al., 2025), anti-inflammatory (Zotsenko et al., 2021), and antimicrobial effects, were not assessed. These activities may, however, be addressed in future studies to provide a more comprehensive evaluation of the pharmacological potential of *Elsholtzia* species.

## 5 Conclusion

This ethnobotanical investigation of *Elsholtzia* species in upper northern Thailand reveals the profound traditional knowledge and cultural importance of these aromatic plants among diverse ethnic groups, including the Akha, Hmong, Karen, Lawa, Lisu, Tai Lue, and Tai Yai. For generations, these communities have utilized *Elsholtzia* species for a wide range of purposes–medicinal, culinary, and as natural insect repellents–demonstrating both the versatility of these plants and their vital role in sustaining local health and livelihoods.

The traditional applications of *Elsholtzia* reflect a sustainable human–environment relationship and highlight the value of indigenous knowledge systems in guiding modern scientific discovery. As contemporary pharmacological studies increasingly validate their anti-inflammatory, antimicrobial, and antiglycation properties, *Elsholtzia* species emerge as promising candidates for further development in pharmaceutical and nutraceutical industries.

The observed variation in the quantity of bioactive compounds and antiglycation activity among specimens collected from different localities underscores the influence of cultivation area and plant part on phytochemical composition. Such variability emphasizes the need for optimized cultivation practices and targeted utilization of plant materials to maximize therapeutic potential.

Overall, the integration of ethnobotanical insights with chemical analysis in this study underscores the interconnectedness of cultural and biological diversity. The findings contribute not only to the documentation of traditional knowledge but also to the advancement of sustainable resource management and drug discovery. Future research should expand on these results by examining large-scale cultivation, assessing environmental and seasonal influences on phytochemical profiles, and performing bioassay-guided isolation of active compounds. Furthermore, continued collaboration with local communities will be essential to ensure that the benefits of bioprospecting foster equitable and sustainable development while safeguarding both cultural heritage and native plant biodiversity.

## Data Availability

The original contributions presented in the study are included in the article, further inquiries can be directed to the corresponding author.
